# A case of abdominal aortic aneurysm presenting with multiple organs embolization

**DOI:** 10.1186/s40792-024-01999-3

**Published:** 2024-08-26

**Authors:** Shinichi Tanaka, Takahiro Ohmine, Takashi Maeda

**Affiliations:** Department of Surgery, Hiroshima Red Cross Hospital and Atomic-Bomb Survivors Hospital, 1-9-6 Sendamachi, Naka-Ku, Hiroshima City, 730-8619 Japan

**Keywords:** Multiple organs, Embolization, Abdominal aortic aneurysm

## Abstract

**Background:**

Distal embolization as the first manifestation of an abdominal aortic aneurysm (AAA) is relatively rare. AAAs presenting with multiple organs embolization are rarer and serious systemic conditions. There are no reports of life-saving outcomes in patients with AAAs presenting with multiple organs embolization prior to surgery.

**Case presentation:**

A 78-year-old man presented with right acute lower limb ischemia (ALLI) and ischemic enteritis coexisting with an 83-mm AAA with a large mural thrombus. Although the patient underwent successful revascularization with endovascular treatment for right ALLI, the manifestation of ischemia of the calf muscles and infection forced right above-knee amputation. The patient also presented with ischemic colitis that resolved with conservative treatment. After receiving appropriate medical therapy and rehabilitation, the patient was successfully treated with open aortic repair for the AAA.

**Conclusion:**

We successfully performed open aortic surgery for a rare case of AAA presenting with multiple organs embolization.

## Background

Presenting distal embolization as the first manifestation of an abdominal aortic aneurysm (AAA) is relatively rare. A report indicated that 5% of patients presented with distal embolization as the first manifestation of their AAA [1]. AAAs presenting with multiple organs embolization are rarer and serious systemic conditions. Herein, we report a 78-year-old male patient with an AAA who presented with right acute lower limb ischemia (ALLI) and acute ischemic colitis.

## Case presentation

**Fig. 1:**
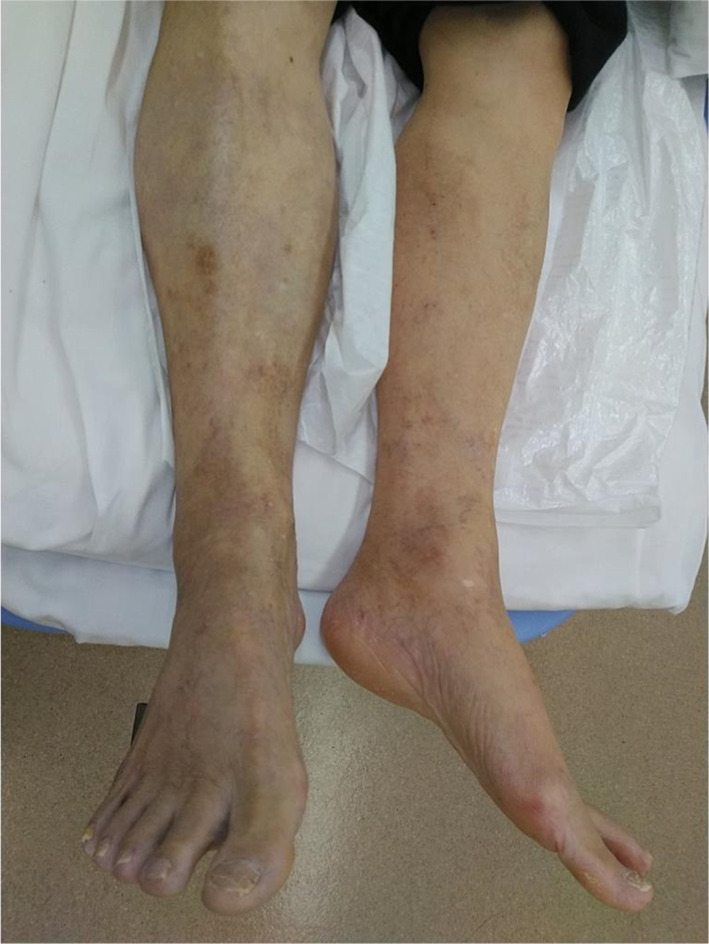
The right leg had prominent coldness and pallor at the time of presentation. The left leg was intact

**Fig. 2:**
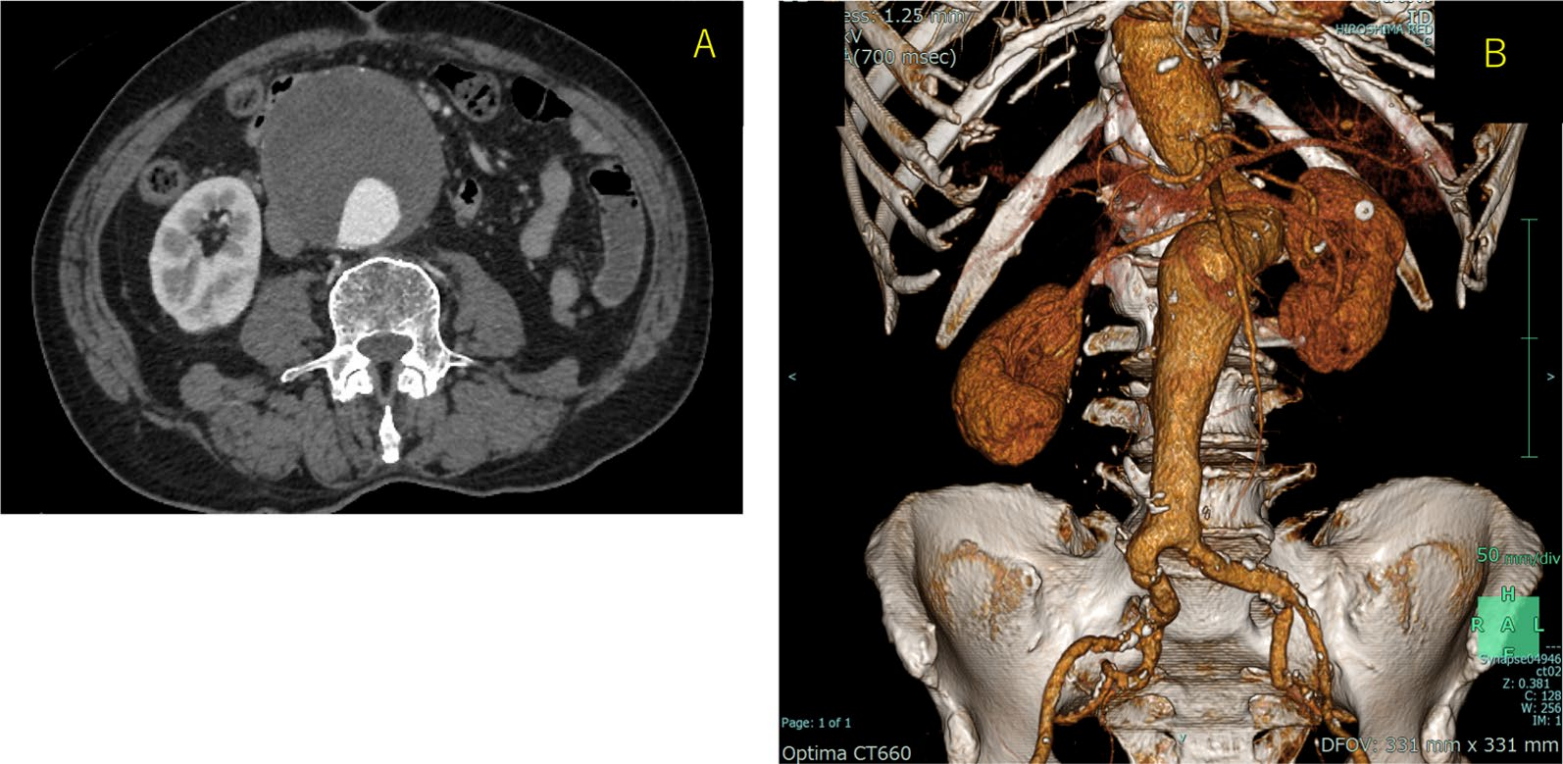
**A** Preoperative enhanced computed tomography (CT) image showing an 83 mm abdominal aortic aneurysm (AAA) with a large mural thrombus. **B** Three-dimensional computed tomography image

**Fig. 3:**
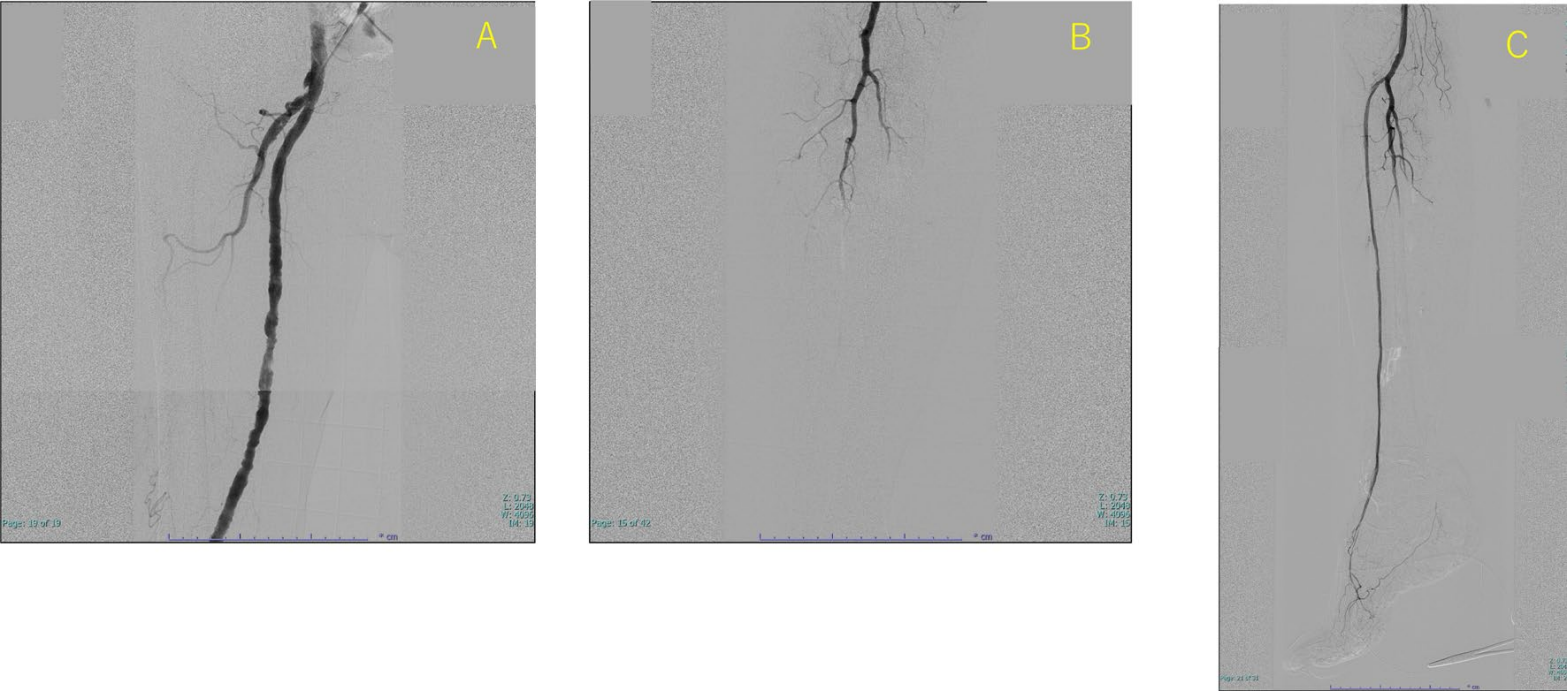
**A** Angiography of the right lower limb revealed no significant stenosis but a circumferential atherosclerotic lesion from the right superficial femoral artery to the popliteal artery. **B** Angiography image showing complete obstruction of all the right tibial arteries. **C** Post-intervention angiography revealed complete recanalization of the right anterior tibial artery and dorsalis pedis artery

**Fig. 4:**
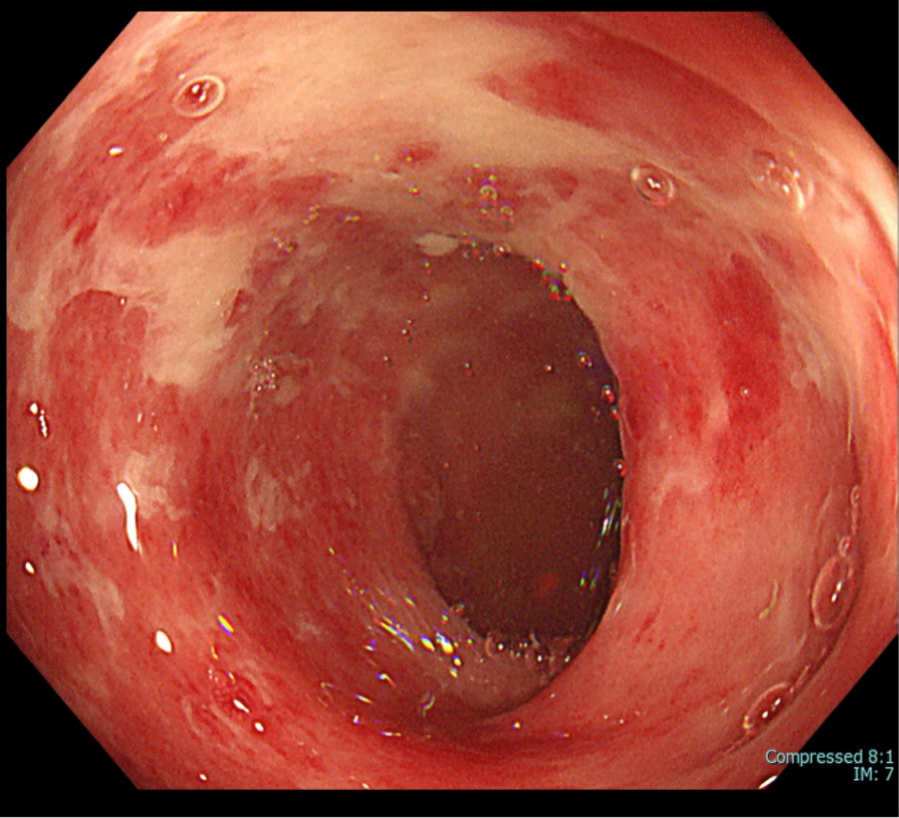
Lower endoscopy image showing multiple longitudinal ulcers from the descending colon to the sigmoid colon, suggesting ischemic colitis

**Fig. 5:**
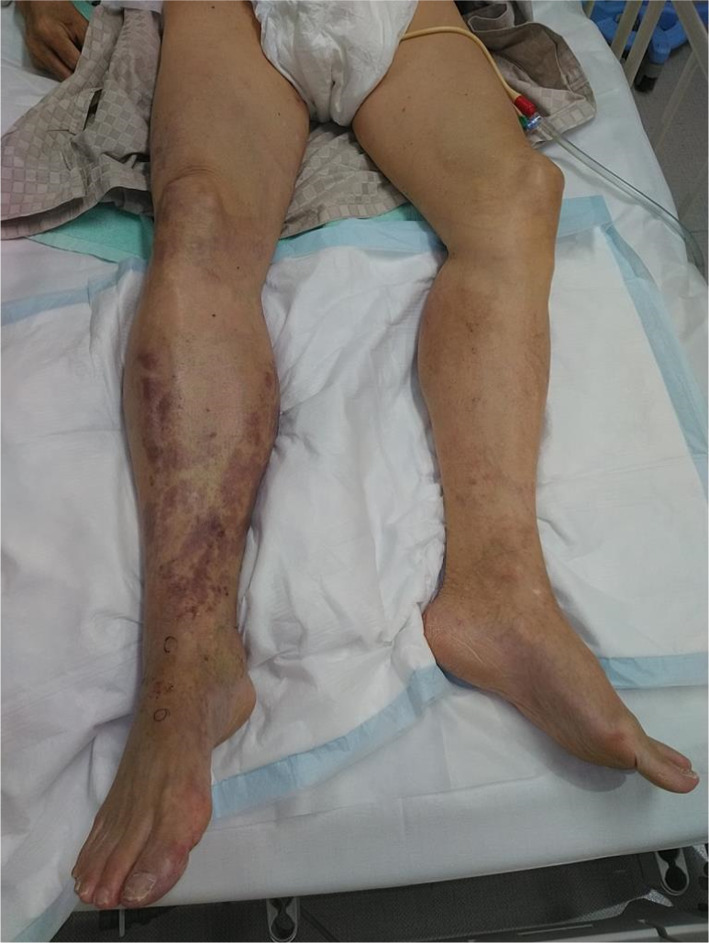
The right leg had blister formation below the knee entirely

**Fig. 6:**
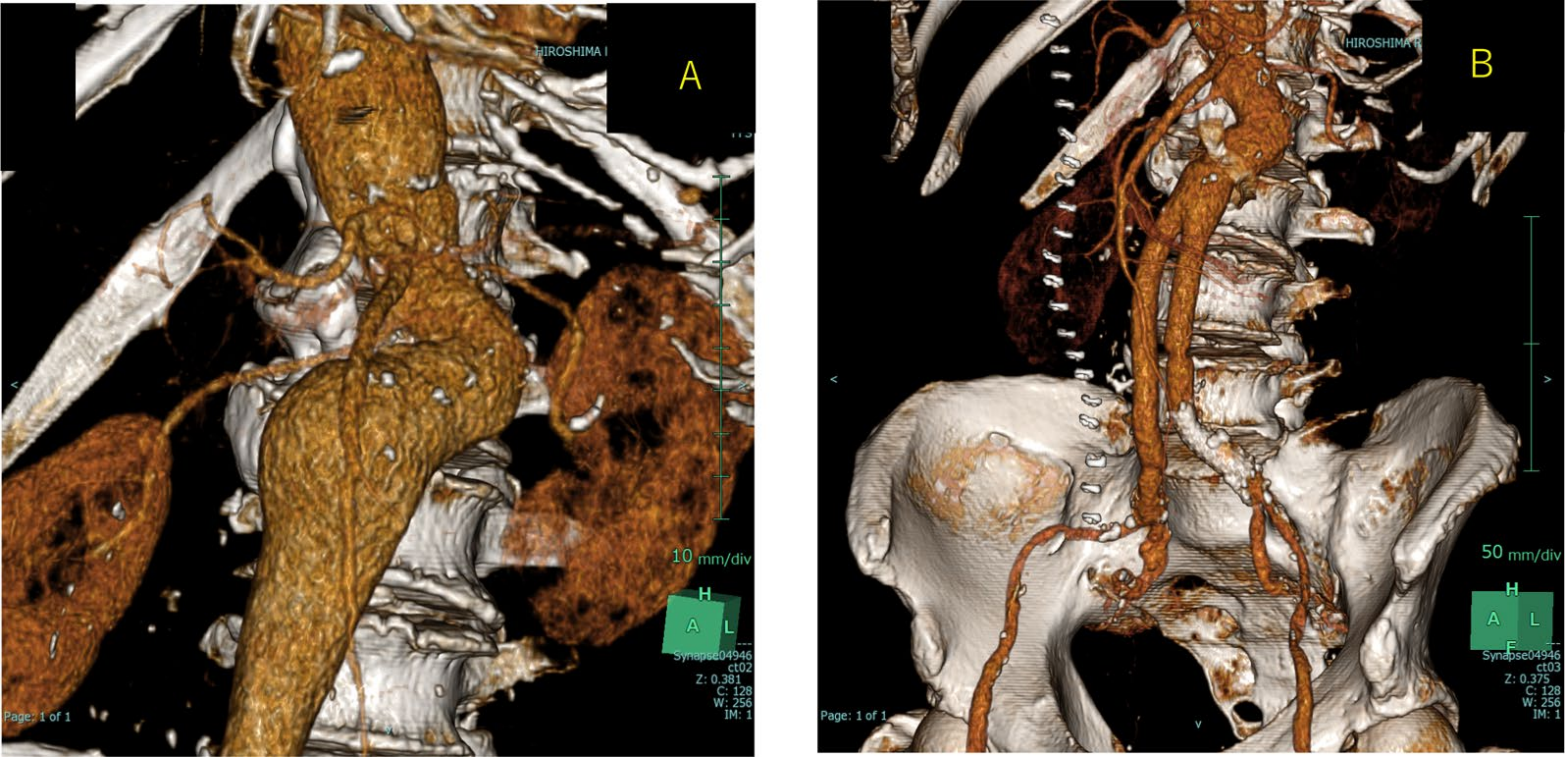
**A** Preoperative three-dimensional computed tomography (CT) image showing the severe proximal neck of the AAA. **B** Postoperative three-dimensional CT image showing the exclusion of the aneurysm and the preservation of the bilateral renal arteries in the patent graft

A 78-year-old man who presented with acute onset of right leg pain, coolness, weakness, and numbness ~ 10 h after the onset of symptoms arrived at our hospital via an ambulance. His past medical history was not relevant other than hypertension. The patient was a current smoker. The right foot exhibited cyanosis, and the patient experienced paresthesia (Fig. 1). Muscle weakness in the dorsiflexion and plantar flexion movements of the right ankle joint was moderate–severe. Although arterial pulsation of the right groin and popliteal fossa was confirmed, peripheral pulses were absent. Normal pulses were palpable in the contralateral leg. No Doppler sound was detected in the right leg. The patient was diagnosed with right acute lower limb ischemia (ALLI), which was classified according to the Rutherford category as Class II-b and III. The electrocardiogram revealed sinus rhythm and complete right bundle branch block. His echocardiogram revealed no thrombus, valvular disease, or shunt. Urgent computed tomography angiography revealed an abdominal aortic aneurysm (AAA) measuring 83 mm in diameter with a large mural thrombus (Fig. 2A, B). Urgent angiography of the right lower limb revealed no significant stenosis but indicated a circumferential atherosclerotic lesion from the right superficial femoral artery to the popliteal artery and complete obstruction of the right tibial vessels (Fig. 3A, B). The anterior and posterior tibial arteries could not cross with only the antegrade wire. The retrograde approach was advanced through the dorsalis pedis artery smoothly. The reconstruction of the right anterior tibial artery by balloon angioplasty was successful (Fig. 3C). Aspirin (100 mg/day) was prescribed after endovascular treatment for ALLI. After successful revascularization, myorenal metabolic syndrome associated with reperfusion was observed. Although the patient presented temporary rapid worsening of renal function, he improved with intravenous infusion only, without dialysis. Two days after revascularization for the right lower limb, the patient complained of acute abdominal pain and bloody stools. The patient was diagnosed with ischemic enterocolitis via lower gastrointestinal endoscopy (Fig. 4). The patient was treated conservatively with blood transfusion and statin induction. His right lower limb showed completely irreversible ischemic changes in calf muscles, although good blood flow through the anterior tibial artery was maintained (Fig. 5). Since limb salvage was impossible due to concomitant infection, above-knee amputation was performed under general anesthesia. The postoperative course was good. Rehabilitation enabled the patient to transfer to a wheelchair on his own, and his daily living activities increased markedly. One month after major amputation, the patient underwent open aortic repair because of the unfavorable anatomical characteristics of the proximal neck (Fig. 6A) and a large mural thrombus for endovascular aortic aneurysm repair. Open aortic repair was performed via a transperitoneal approach. The left renal vein was divided close to the inferior vena cava. Bilateral infrarenal clamping was performed for proximal aortic exposure. Complete repair was performed using a bifurcated graft (Hemashield Platinum 22–11 mm, Boston Scientific, Marlborough, MA, USA) (Fig. 6B). The postoperative course was uneventful. He was transferred for continuing rehabilitation 1 month postoperatively.

## Discussion

The majority of abdominal aortic aneurysms (AAAs) are asymptomatic and are detected as incidental findings by imaging performed for other purposes. Presenting distal embolization as the first manifestation of AAA is relatively rare [1]. AAAs presenting with multiple organs embolization are thought to be rarer and serious systemic conditions. To the best of our knowledge, there are few case reports in English concerning surgical salvage of AAAs presenting with multiple organs embolization.

The etiologies of acute lower limb ischemia (ALLI) are arterial embolism, atherosclerotic/in situ thrombosis, multifactorial factors and stent- or graft-related thrombosis [2]. In actual clinical practice, accurately determining the etiology of ALLI is often difficult. Indeed, the involvement of in situ thrombosis cannot be completely ruled out in our case. Furthermore, this patient may have experienced both thrombosis and embolism; thus, this etiology could be multifactorial. However, when this case of ALLI was examined comprehensively, embolism was most likely involved.

Ischemic colitis is a generally known complication after AAA repair. Before surgery for AAA, the patient presented with ischemic colitis. The mechanism of this case was thought to involve the dislodging of thromboemboli or atheroemboli from the AAA to the distal mesenteric arteries, resulting in the development of ischemic colitis. The lower gastrointestinal endoscopy results indicate that embolism most likely occurred at the inferior mesenteric artery, which primarily supplies the descending colon to the sigmoid colon. Because the onset of ischemic colitis was 2 days after the introduction of aspirin, distal embolism could relate to new-start of aspirin.

Statins have known to reduce low-density lipoprotein cholesterol. Pleiotropic effects of statins such as influencing endothelial function and plaque stability could affect perioperative outcomes after cardiovascular surgery [3]. Further randomized trial studies are needed to determine the effect of statins in open aortic repair, but previous studies have shown better perioperative outcomes of AAA repair with statin therapy [4, 5] Retrospective study data support a beneficial role of statin use prior to surgery for patients undergoing endovascular aortic repair, decreasing the incidence of lower extremity embolic complications [4]. However, in open aortic repair, statin does not significantly reduce the incidence of lower extremity embolic complications [4]. Another study revealed that statin therapy was independently associated with improved long-term survival after either open aortic repair or endovascular aortic repair [5].

No further distal emboli have occurred since the introduction of medical therapy and rehabilitation intervention. In our case, endovascular aortic repair was not indicated to avoid further embolization, and open aortic repair was preferred. The patient’s background of multiple organs embolization and postoperative state of major amputation made open aortic repair more invasive, but appropriate medical therapy and rehabilitation may prevent further cardiovascular complications and reduce perioperative mortality.

## Conclusion

We report a successful surgical salvage case of abdominal aortic aneurysm presenting with multiple organs embolization.

## Data Availability

Not applicable.

## Abbreviations

AAA: Abdominal aortic aneurysm
ALLI: Acute lower limb ischemia
